# Sprouting and network data assessed in the chicken aortic ring assay under stimulation with human adipose-derived stem cell secretomes harvested from single cells or spheroids of different sizes

**DOI:** 10.1016/j.dib.2025.111371

**Published:** 2025-02-05

**Authors:** Petra Wolint, Silvan Hofmann, Julia von Atzigen, Roland Böni, Iris Miescher, Pietro Giovanoli, Maurizio Calcagni, Maximilian Y. Emmert, Johanna Buschmann

**Affiliations:** a Division of Surgical Research, University Hospital of Zurich, 8091 Zurich, Switzerland; b Department of Plastic Surgery and Hand Surgery, University Hospital Zurich, 8091 Zurich, Switzerland; c White House Center for Liposuction, 8044 Zurich, Switzerland; d Institute for Regenerative Medicine (IREM), University of Zurich, 8952 Zurich, Switzerland; e Deutsches Herzzentrum der Charité (DHZC), Department of Cardiothoracic and Vascular Surgery, 13353 Berlin, Germany; f Charité-Universitätsmedizin Berlin, Corporate Member of Freie Universität Berlin and Humboldt-Universität zu Berlin, 10117 Berlin, Germany; g BIH Center for Regenerative Therapies (BCRT), Berlin Institute of Health at Charité-Universitätsmedizin Berlin, 13353 Berlin, Germany

**Keywords:** Angiogenesis, Aortic ring assay, Secretome, Mesenchymal stem cells, Spheroid, Chicken

## Abstract

The aortic ring assay is widely used to assess the angiogenic modulation evoked by various drugs. However, different parameters are used as readouts and there is no congruence in terminology. In addition, some researchers use one set of parameters, and other researchers choose parameters that overlap to some extent, but miss a part of these parameters, rendering a direct comparison of the readouts and results difficult [1]. Therefore, we present data acquired in the chicken aortic ring assay that cover the full spectrum of possible readouts – exemplified by stimulation with secretomes harvested from human adipose-derived stem cells, cultivated either as single cell monolayer culture or as 3D spheroids, sized 250 cells/spheroid or 8000 cells/spheroid, respectively.

## Graphical abstract

**Figure:**
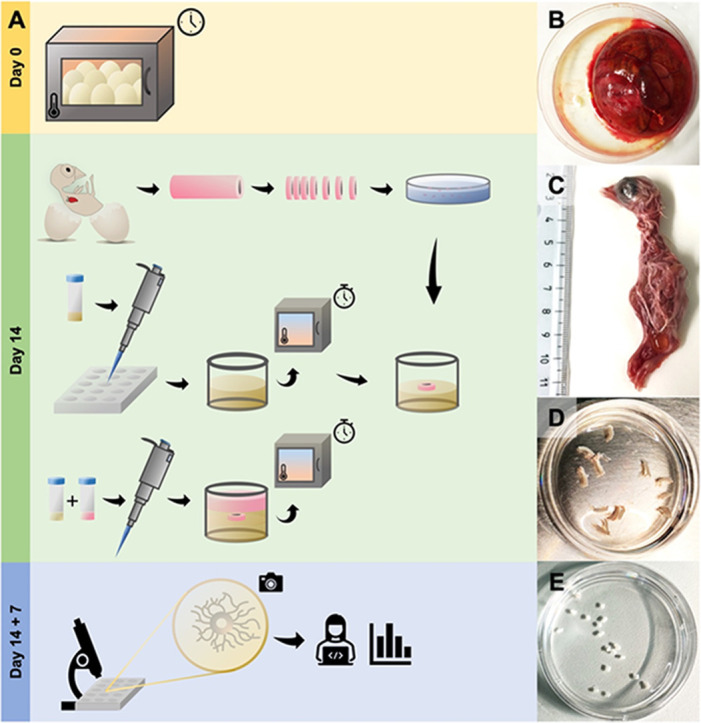

Specifications Table

**Table:** 

Subject	Angiogenesis, cell biology
Specific subject area	Cell culture data were determined for monolayer human adipose-derived stem cell culture or by using the same cells cultured as spheroids with 250 or 8000 cells/spheroid, respectively (n = 3 donors for each format). The secretomes were applied on the chicken aortic ring assay and parameters were assessed on days 1, 4 and 7, respectively.
Data format	The data consist of raw and analysed data.
Type of data	Excel files of the type *.xlsx* files.
Data collection	Chicken aortas were carefully removed from embryos at incubation day 14. The aortas were cut in pieces, yielding rings of 1 mm thickness. These rings were transferred into matrigel containing plates where during the first four days secretomes harvested from human adipose-derived stem cells were applied; and microscopic images were taken on days 1, 4, and 7, respectively. The secretomes were harvested from single cell monolayer culture; from spheroids with 250 cells/spheroid and from spheroids with 8000 cells/spheroid.
Data source location	All data were collected in Zurich, Switzerland, at the University Hospital Zurich in the laboratories of the Department for Plastic Surgery and Hand Surgery.
Data accessibility	**Repository name**: Mendeley Data **Data identification number**: DOI:10.17632/ryzvs2zw9v.1 **Direct URL** to data: Network characterization data assessed in the chicken aortic ring assay using human adipose-derived stem cell secretomes - Mendeley Data
Related research article	Standardization to Characterize the Complexity of Vessel Network Using the Aortic Ring Model

## 1 Value of the Data

• Our data can be reused, if other human cell types than human adipose-derived stem cells are used to harvest secretome.
• These data can be reused, if other species than human are used for the extraction of adipose-derived stem cells and to produce secretomes from them.
• These data can be compared with drugs that are supplemented to the chicken aortic ring assay.
• Our data may act as reference data for aortic rings collected from pig, horse, rat or other animals than chicken.
• These data can be reused for comparison with secretomes harvested from spheroids having other sizes than 250 or 8000 cells per spheroid, respectively.

## 2 Background

Angiogenesis is a major process in mammalian life and is extensively studied in vitro and in vivo, because it is involved in many kinds of diseases [2,3]. Proper models to study angiogenesis are therefore very important, at best providing well-defined parameters as readouts and a congruent terminology. The aortic ring assay is one model out of many which is widely used to test drugs [4], however, parameters assessed vary from study to study and are often named differently; depending on the research community using them in different contexts. Hence, we provide a comprehensive spectrum of different parameters that can be quantified with the aortic ring assay. We describe according data assessed in the aortic ring assay, using aorta from the chicken embryo, and stimulating the network sprouting by three kinds of stem cell secretome, harvested from human adipose-derived stem cells cultivated as single cells, 250 cells/spheroid or 8000 cells/spheroid. The secretomes have the potential to modulate angiogenesis and therefore are convenient to generate differential data in the chicken aortic ring assay.

## 3 Data Description

The data are stored as a Microsoft Excel (Microsoft Corporation, Redmond, WA, USA) file (.xlsx file) in a Mendeley Data repository service entitled Network characterization data assessed in the chicken aortic ring assay using human adipose-derived stem cell secretomes - Mendeley Data.

This repository contains six excel files, named DiB_Figure_1_manuscript vessel network complexity; DiB_Figure_2_manuscript vessel network complexity; DiB_Figure_3_manuscript vessel network complexity; DiB_Figure_4_manuscript vessel network complexity; DiB_Figure_S2_manuscript vessel network complexity; and DiB_Table_S1_manuscript vessel network complexity, respectively. The titles of these excel files contain the *Figure* number of the related research article, to assure where which of the raw data have been analysed to generate the corresponding figures [1].

### 3.1 Matrix used for the aortic ring assay

**File** DiB_Figure_1_manuscript vessel network complexity

This excel file is open access published in Mendeley Data Network characterization data assessed in the chicken aortic ring assay using human adipose-derived stem cell secretomes - Mendeley Data and contains 3 sheets, called *Fig1E, Fig1F*, and *Fig1G*, respectively, according to the panels in the related research article Figure 1 [1]. The first sheet *Fig1E* contains the vessel area data in mm^2^, assessed for four matrices; egg white, bovine collagen, jellagel and Matrigel®. The second sheet *Fig1F* contains the maximum outgrowth radius in μm, also assessed for four matrices; egg white, bovine collagen, JellaGel^TM^ and Matrigel®. The third sheet *Fig1G* contains the number of endpoints per aortic ring for the four matrices; egg white, bovine collagen, JellaGel^TM^ and Matrigel®.

### 3.2 Embryos, spheroids and secretomes

**File** DiB_Figure_2_manuscript vessel network complexity

This excel file is open access published in Mendeley Data Network characterization data assessed in the chicken aortic ring assay using human adipose-derived stem cell secretomes - Mendeley Data and contains 7 sheets, called *Fig2D, Fig2E, Fig2F, Fig2G, Fig2H, Fig2I*, and *Fig2J*, respectively, according to the panels in the related research article Figure 2 [1]. The first sheet *Fig2D* contains the diameter of the spheroids in μm for spheroids large = 8000 cells/spheroid in column D and for spheroids small = 250 cells/spheroid in column H. The second sheet *Fig2E* contains total protein concentrations in μg/mL, assessed for the three experimental secretome groups – single cells, spheroids small and spheroids large, as well as for serum free medium, respectively (column B). In column D, the average protein concentrations as assessed for serum free medium was subtracted from column B, resulting in the protein concentration evoked by the cells under view. Finally, in column E, the value obtained in column D, was divided by 10 to achieve the concentration of the proteins in the corresponding secretomes.

The third sheet *Fig2F* contains data on the weight of the embryos in g, with mean and standard deviation (SD). The fourth sheet *Fig2G* contains data on the circumference of the aortic rings used in the experiments in μm, assessed for control rings and the three experimental groups; single cells, spheroids small and spheroids large, respectively. Then, the fifth sheet *Fig2H* contains data on the percentage of migration for days 1, 4 and 7. *Fig2I* is the name of the sixth sheet that contains data on primary initial vessels in relation to total vessel number in %. Finally, the sheet *Fig2J* contains data on number of loops per aortic ring for the four experimental groups (line 1) and for the three different time points (line 2).

### 3.3 Network characterization

**File** DiB_Figure_3_manuscript vessel network complexity

This excel file is open access published in Mendeley Data Network characterization data assessed in the chicken aortic ring assay using human adipose-derived stem cell secretomes - Mendeley Data and contains 6 sheets, called *Fig3A, Fig3B, Fig3C, Fig3D, Fig3E,* and *Fig3F*, respectively, according to the panels in the related research article Figure 3 [1]. Sheet *Fig3A* contains data on maximum radial outgrowth in μm, sheet Fig3B contains data on maximum initial vessel length in μm, sheet Fig3C contains data on the total vessel length in μm, sheet Fig3D contains data mean initial vessel length in μm, sheet *Fig3E* contains data on the speed mean vessel length in μm/day and sheet *Fig3F* provides data on the speed maximum vessel length in mm/day, respectively. For all sheets, the four experimental groups are denoted in line 1 and the three different time points in line 2. Additionally, below, the mean and the standard deviation are calculated.

### 3.4 Sprouting

**File** DiB_Figure_4_manuscript vessel network complexity

This excel file is open access published in Mendeley Data Network characterization data assessed in the chicken aortic ring assay using human adipose-derived stem cell secretomes - Mendeley Data and contains 6 sheets, called *Fig4A, Fig4B, Fig4C, Fig4D, Fig4E,* and *Fig4F*, respectively, according to the panels in the related research article Figure 4 [1]. Sheet *Fig4A* contains data on the vessel density in numbers of vessels per mm of the aortic ring, while sheet *Fig4B* presents data on the number of branches. Sheet *Fig4C* contains data on the mean branch length in μm, while sheet *Fig4D* has data on the relative frequency of branch lengths (in %). As for *bin* center in field A1, it is defined as follows: “each bin contains the number of values that lie within the range of values that define the bin” (https://www.graphpad.com/guides/prism/latest/statistics/stat_howto_frequency_distribution.htm). The sheet *Fig4E* then represents data on the number of junctions, while the sheet *Fig4F* contains data on the number of junctions per initial vessels.

### 3.5 Angiogenic activity index

**File** DiB_Figure_S2_manuscript vessel network complexity

**File** DiB_Table_S1_manuscript vessel network complexity

The first excel file is open access published in Mendeley Data Network characterization data assessed in the chicken aortic ring assay using human adipose-derived stem cell secretomes - Mendeley Data and contains 1 sheet, called *FigS2*. It contains data for all parameters, all subindices and the angiogenic activity index – for the three donors separately. The second excel file is open access published in Mendeley Data Network characterization data assessed in the chicken aortic ring assay using human adipose-derived stem cell secretomes - Mendeley Data and contains 1 sheet, called *TabS1*, which contains data for all parameters, all subindices and the angiogenic activity index, averaged for the three donors. In column B, a predictive importance factor is given, which is either 1 or 2, and indicates how much a parameter is weighted (1 = normal; 2 = much) and hence there are two options for each experimental group, either unweighted with a predictive importance factor = 1 (columns C, E and G), or weighted with a predictive importance factor = 2 (columns D, F and H).

## 4 Experimental Design, Materials and Methods

### 4.1 Secretome

In accordance with the local Ethics Committee of the Canton Zurich in Switzerland and with a licence from this committee (KEK-ZH-Nr. 2010-0476/0), lipoaspirates from three human patients were collected after their written consent and used for the isolation of adipose-derived stem cells (ASCs). These cells were either cultured as single cell culture in monolayers; or as small (250 cells/spheroid) and large (8000 cells/spheroid) spheroids according to a protocol in our related research article [1]. After harvesting the secretomes, they were concentrated 10 times to assess the total protein content according to a detailed description in the related research article [1].

### 4.2 Chicken aortic ring assay

The chicken aortic ring assay was performed as described previously [1]. Briefly, on incubation day 14, the embryos of fertilized Lohmann white LSL chicken eggs (Animalco AG Geflügelzucht, Staufen, Switzerland) were opened at the chest, the heart carefully removed, and the aorta exposed and harvested. Next, the aorta was sliced into 1 mm thick rings, which were then transferred to plates containing matrigel, the matrix that showed to be superior to egg white, jellagel or bovine collagen, respectively. The rings were covered with 50 μL of matrigel, before the experiment started by dropping 30 μL of secretome to the system every day during the first four days. On days 1, 4 and 7, microscopic imaging was performed for longitudinal documentation of sprouting and network (Zeiss Axio Vert.A1 brightfield microscope with ZEN 2.6 lite software from Carl Zeiss Microscopy, Germany).

### 4.3 Categories and parameter definitions

The microscopic images were analysed for a comprehensive set of parameters that are defined in Table 1.

**Table 1:** Categories and parameters used to analyse the microscopic images taken in the chicken aortic ring assay.

Parameter (Category)	Definition
Migration of explant (explant)	Migration of the explant was given when sprouting became visible.
Circumference (explant)	Measurement of how long was the outer circumference of the explant.
Vessel structure (pattern)	The proportion of initial vessels compared to existing branches was determined for vessel structure.
Number of loops (pattern)	The loops were counted. Definition of loop: Three or more branches interconnected forming a circle.
Maximum radial outgrowth (network)	Length measured at a 90° angle from the outer edge of the ring to the tip of the longest vessel.
Maximum initial vessel length (network)	Length measured from the outer edge of the ring without taking possible branches into account – not necessarily at a 90°C angle.
Total vessel length (network)	Sum of all initial vessels and branches.
Mean total vessel length (network)	Mean calculated based on all vessels and branches.
Mean and maximum vessel length per hour and per day (network)	Using the exact time that elapsed between taking the microscopic images on days 4 and 7, the speed was calculated on how much vessels have grown.
Vessel count (sprouting)	Number of initial vessels determined without branches.
Number of branches (sprouting)	Number of individual branches.
Mean branch length (sprouting)	Averaged length of the branches.
Number of junctions (sprouting)	If at least one branch originated from an initial vessel. The number of junctions was expressed as a number of junctions and as junctions per vessel.
Angiogenic Activity Index (all categories)	Angiogenic activity index (AAI) = $\frac{\left\{AR^{7thDoT}\left[Treatment\right]-AR^{4thDoT}\left[Treatment\right]\right\}-\left\{AR^{7thDoT}\left[Control\right]-AR^{4thDoT}\left[Control\right]\right\}}{\left\{AR^{7thDoT}\left[Control\right]-AR^{4thDoT}\left[Control\right]\right\}}$
AR (all categories)	**A**ngiogenic **R**esponse of the respective examined parameter
DoT (all categories)	**D**ay **o**f **T**reatment with the secretome group (Treatment) or serum-free medium group (Control).
AAI for subindex	AAI for subindex = $\frac{1}{n}\sum_{i=\;1}^{n}AAI_{i}$
Final AAI	Final AAI = $\frac{1}{n}\sum_{i=\;1}^{n}SI_{i}$

## Limitations

The chicken aortic ring assay was performed for 7 days and images were taken at 1, 4 and 7 days. One could extend the whole experiment to longer periods of time. Also, it would be possible to monitor vessel and sprouting development in timely narrower intervals, i.e. daily taking photos; enabling to calculate an AAI as a function of time.

## Ethics Statement

The study was conducted in accordance with the Declaration of Helsinki and approved by the Ethics Committee of the Cantonal Ethics Committee Zurich in Switzerland (KEK-ZH-Nr. 2010-0476/0).

## CRediT authorship contribution statement

**Petra Wolint:** Conceptualization, Methodology, Data curation, Investigation, Writing – original draft, Writing – review & editing. **Silvan Hofmann:** Conceptualization, Methodology, Data curation, Investigation, Writing – original draft, Writing – review & editing. **Julia von Atzigen:** Data curation, Writing – review & editing. **Roland Böni:** Data curation, Writing – review & editing. **Iris Miescher:** Data curation, Writing – review & editing. **Pietro Giovanoli:** Supervision, Writing – review & editing. **Maurizio Calcagni:** Supervision, Writing – review & editing. **Maximilian Y. Emmert:** Supervision, Funding acquisition, Writing – review & editing. **Johanna Buschmann:** Conceptualization, Supervision, Writing – original draft, Writing – review & editing, Funding acquisition, Project administration.

## Data Availability

Mendeley DataNetwork characterization data assessed in the chicken aortic ring assay using human adipose-derived stem cell secretomes (Original data).

## References

[1] Wolint P., Hofmann S., von Atzigen J., Böni R., Miescher I., Giovanoli P., Calcagni M., Emmert M.Y., Buschmann J. (2025). Standardization to characterize the complexity of vessel network using the aortic ring model. Int. J. Mol. Sci..

[2] Griffioen A.W., Dudley A.C. (2022). The rising impact of angiogenesis research. Angiogenesis.

[3] La Mendola D., Trincavelli M.L., Martini C. (2022). Angiogenesis in disease. Int. J. Mol. Sci..

[4] Mehta V., Mahmoud M. (2022). Ex vivo mouse aortic ring angiogenesis assay. Methods Mol. Biol..

